# Image-Guided TIVAP by Interventional Radiologists: A Single-Center Cohort Study Using Institutional Surgical Benchmarks

**DOI:** 10.3390/jcm15218562

**Published:** 2026-11-03

**Authors:** Nour Maalouf, Mazen Abou Mrad, Fiona Mankertz, Reza Dehdab, Isabelle Schöpper, Rosa Alba Pugliesi, Saif Afat, Jonas C. Apitzsch

**Affiliations:** 1 Department of Radiology, Tübingen University Hospital, 72076 Tübingen, Germany; 2 Department of Anesthesiology and Intensive Care, Tübingen University Hospital, 72076 Tübingen, Germany; 3 Department of Diagnostic and Interventional Radiology, Krankenhaus Porz am Rhein, 51149 Köln, Germany; 4 Section of Radiology, Department of Biomedicine, Neuroscience and Advanced Diagnostics (BiND), University of Palermo, Via del Vespro 129, 90127 Palermo, Italy; 5 Department of Radiology and Nuclear Medicine, Helios Hospital Pforzheim, 75175 Pforzheim, Germany

**Keywords:** vascular access devices, central venous, interventional, health care costs, health resources

## Abstract

**Background/Objectives**: To evaluate the resource utilization, procedural costs, procedure time, and intraprocedural outcomes of interventional radiology (IR)-guided totally implantable venous access port (TIVAP) placement and contextualize these findings using institutional surgical benchmarks. **Methods**: We conducted a single-center study between March 2020 and March 2024 that included 93 patients requiring TIVAP placement for chemotherapy. All ports were inserted under ultrasound and fluoroscopic guidance in an angiography suite. Surgical insertions using a cephalic vein cut-down under anesthesia were used as a comparator. Data collected included number of consent forms, staffing, procedure duration, material and medication utilization, and procedural costs obtained from hospital financial records. Descriptive statistics were used to summarize outcomes. **Results**: IR-guided TIVAP insertion required one combined consent form and two personnel, compared with two consent forms and four personnel for the surgical workflow. The average procedural cost in the angiography suite was €168.16 versus an institutional surgical benchmark of €407.90, representing a 58.8% lower observed cost. The recorded average procedure time was 45 min for IR versus 85 min for the surgical benchmark. Material costs were comparable, except for intravenous anesthetic agents used in the operating room. No intraprocedural complications occurred among the 93 IR cases. **Conclusions**: IR-guided TIVAP implantation required fewer personnel and was associated with lower observed procedural costs and shorter recorded procedure times relative to institutional surgical benchmarks. The absence of a patient-level surgical comparator and long-term follow-up limits conclusions regarding comparative safety and clinical outcomes.

## 1. Introduction

Totally implantable venous access ports (TIVAPs), commonly called chemoports, are widely used in oncology for reliable long-term venous access. Compared to peripheral intravenous lines, TIVAPs are associated with lower overall complication rates such as fewer infections and thrombotic events as well as improved patient quality of life [1,2,3]. As they are fully implanted beneath the skin, ports enable patients to carry out daily activities, such as swimming and other water sports, with greater comfort and fewer limitations compared to external catheters [2]. Traditionally, port insertion is performed by surgeons in an operating theater, typically under sedation with propofol, and in some cases—for example, in patients with tomophobia—under general anesthesia [4,5]. However, recent studies have shown a rise in central venous port systems implanted percutaneously by interventional radiologist [6,7]. The development of interventional radiology techniques has enabled percutaneous port implantation under imaging guidance as an alternative. The Seldinger technique with ultrasound-guided venous access allows radiologists to place ports without a surgical cut-down, whereas the surgical approach often involves a cut-down on the cephalic or subclavian vein using anatomical landmarks and fluoroscopy for catheter confirmation [8].

Over the past two decades, numerous studies have demonstrated the feasibility and safety of ports placed by interventional radiologists. For example, Lorch et al. reported successful placement of 125 ports by IR with excellent outcomes [5]. Likewise, a large single-center series by Yaacob et al. achieved a 99.4% technical success rate for 161 IR-guided port insertions, with minimal immediate complications; only 1.2% of cases incurred an arterial puncture and there was no incidence of pneumothorax [6]. These and other reports reflect an increasing utilization of image-guided port placement in clinical practice [9,10,11]. Internal jugular vein access under ultrasound guidance has become a preferred method in IR as it avoids the need for a surgical incision and has shown advantages such as lower pain and complication rates compared to subclavian vein access [12]. Both surgeons and interventional radiologists are technically capable of performing port implantation; however, the overall resource utilization and efficiency of the two approaches may differ significantly.

In our institution, we observed an anecdotal shift during the COVID-19 pandemic where more port insertions were handled by IR services, prompted by operating room constraints and anesthesia staffing challenges. This study was conducted to quantitatively compare the two approaches, surgical versus IR-guided port insertion, in a controlled setting, focusing on factors such as personnel involved, procedure time, material and medication usage, costs, and patient outcomes. We hypothesized that IR-guided port placement offers efficiency gains and cost savings without compromising safety or effectiveness. The results of this analysis could inform hospital policies and allocation of port insertion cases to the most suitable clinical service.

## 2. Materials and Methods

**Study Design and Patient Selection:**

We conducted a single-center study of TIVAP insertions performed by interventional radiologists in the angiography suite with over 15 years of experience in the field, with outcomes benchmarked against institutional data for insertions performed by surgeons in the operating room with over 13 years of experience. From March 2020 to March 2024, 93 adult patients who underwent IR-guided port placement were included. All patients had a clinical indication for a totally implantable venous access port for chemotherapy administration. Exclusion criteria were limited to contraindications precluding port insertion and lack of venous access in the upper body. Allocation to the IR or surgical pathway was determined by procedural scheduling and time-slot availability rather than by randomization. Patients were not systematically selected according to BMI, previous neck surgery, or other anatomical characteristics. Patients considered clinically suitable for port implantation under local anesthesia were eligible for the IR pathway. Accordingly, the IR cohort represents a selected patient population and should not be considered clinically equivalent to the historical surgical population. Patient-level data were available for the IR cohort, whereas the surgical benchmark was based on institutional database averages and the standard surgical protocol used at our center. Patient-level matched surgical controls were not available. Written informed consent was obtained from each IR patient, and the study was approved by the institutional review board. During the preparation of this manuscript, the authors used ChatGPT (OpenAI, GPT-5.6) for language editing and to improve the clarity, readability, and presentation of the manuscript. All AI-generated output was reviewed and edited by the authors, who take full responsibility for the content of the publication.

**Interventional Radiology Procedure:**

**Figure 1:**
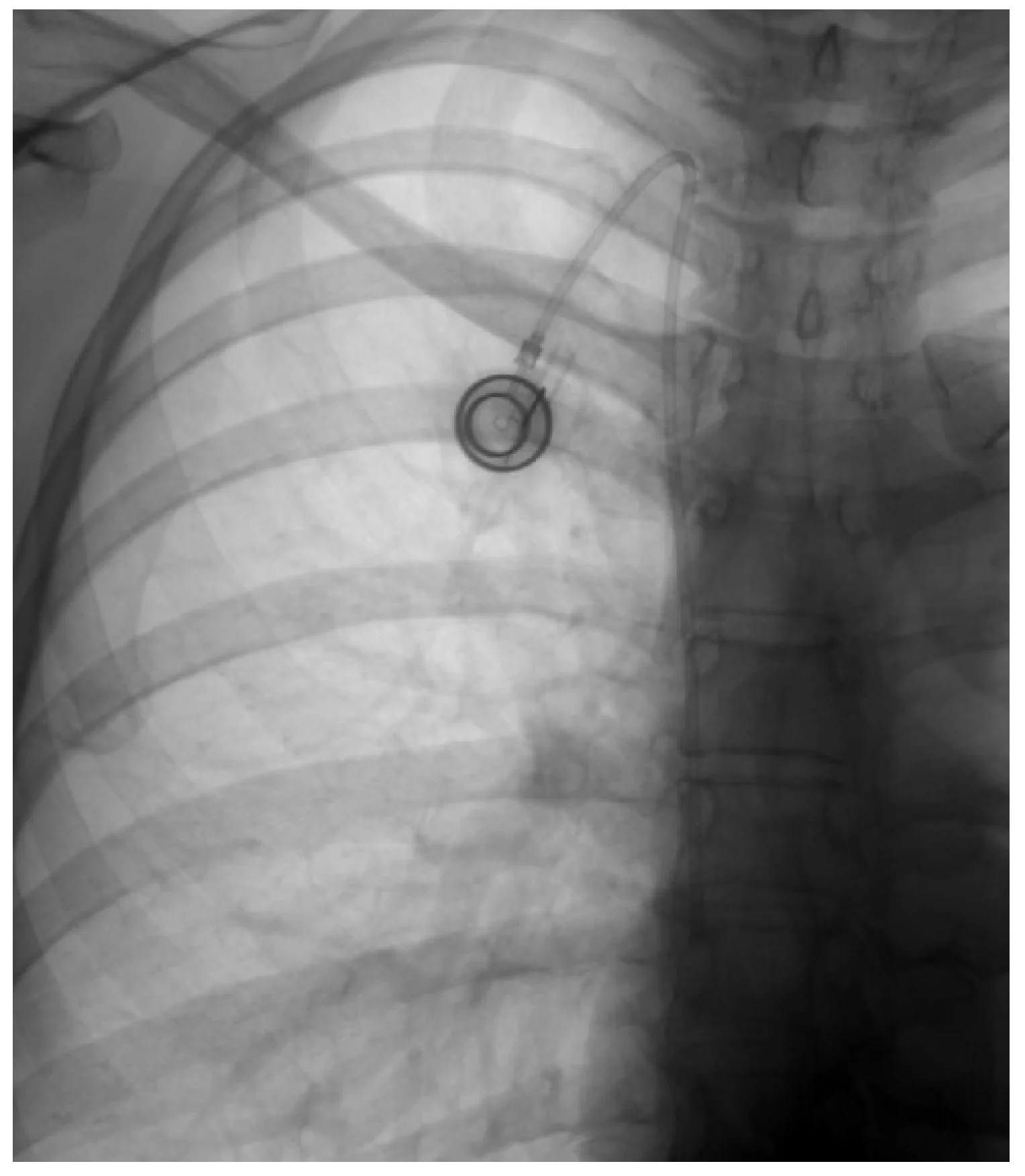
Intraprocedural static fluoroscopic spot radiograph obtained during port implantation in the interventional radiology suite, showing the implanted port chamber, catheter course, and catheter tip position at the cavoatrial junction.

For each patient, the interventional radiologist first verified the indication for port placement and screened for contraindications, including reviewing coagulation parameters such as prothrombin time, aPTT, INR and platelet count. Prior to the planned procedure, the interventional radiologist met with the patient to provide an explanation of the procedure and to obtain single written informed consent for port insertion under local anesthesia. Procedure planning was carried out to ensure that the selected port site would not interfere with concurrent therapy, avoiding placement on the ipsilateral side of the tumor and outside the anticipated radiation field, if radiotherapy was planned. All IR placements were performed in an angiographic suite under sterile conditions. As the first step, a suitable venous access was identified using ultrasound. The skin at the neck and chest was disinfected and draped. Local anesthesia (10 mL of 1% mepivacaine, standardized amount) was infiltrated at the intended venipuncture and port pocket sites. An 18G Abbocath needle (Abbott Laboratories Inc., Abbott Park, Illinois, United States) attached to a saline-filled syringe was used to puncture the vein under ultrasound guidance. After confirmation of venous entry by aspiration of blood, the needle’s inner stylet was removed, leaving the sheath in place. A 0.035-inch guidewire (Terumo™, Tokyo, Japan) was inserted through the sheath and advanced under fluoroscopy into the superior vena cava/right atrium junction. Next, a small skin incision was made at the venipuncture site, and a dilator and introducer sheath were advanced over the wire into the vein. The guidewire and dilator were then removed, and the peel-away introducer sheath was left in place, occluded with a thumb to prevent air entry. Attention was turned to creating the port pocket where a 3 cm incision was made on the anterior chest usually below the clavicle and a subcutaneous pocket was bluntly dissected to accommodate the port reservoir. A small subcutaneous tunnel was created from the pocket to the neck puncture site. The port catheter, pre-flushed with saline and contrast, was then fed through the introducer sheath into the central vein, with its tip positioned at the cavoatrial junction. The latter is then confirmed on fluoroscopy during patient inspiration. The sheath was peeled away, and the catheter was trimmed to an appropriate length and connected to the port chamber. Using the tunnel, the catheter was pulled so that the port chamber sat in the prepared pocket. Two anchoring absorbable sutures were placed through the port’s suture holes and tied to the deep fascial tissue to secure the port’s position. The system was tested by flushing and aspirating through the port, and an X-ray was performed to verify proper catheter tip location and patency (Figure 1). Finally, the pocket and neck incisions were closed starting with deep absorbable sutures and ending with a superficial intradermal stitch. A sterile dressing was applied. All IR procedures were performed with the patient under local anesthesia and optional mild sedation with 1–2 mg oral lorazepam for highly anxious patients. Patients were observed briefly post-procedure and could go home the same day.

**Surgical Procedure:**

In the conventional surgical pathway at our hospital, port insertions are performed by the surgery and anesthesiology teams. Patients first have a preoperative meeting with the surgeon for consent, followed by a separate meeting with an anesthesiologist to evaluate fitness for anesthesia and obtain anesthesia consent. On the day of surgery, standard anesthesia protocols are followed. Typically, light general anesthesia is performed with agents such as propofol or midazolam, and local anesthetic is given at the incision site. After induction of anesthesia and sterile preparation of the chest and neck region, a surgical cut-down technique is often employed where a 3 cm incision is made infraclavicularly to expose the cephalic vein. The vein is isolated and ligated distally, and then a venotomy is performed to insert the catheter. In some cases where the cephalic vein is unsuitable, surgeons may instead puncture the subclavian vein via landmark guidance and thread the catheter via the Seldinger technique. For the surgical technique described in our institution’s protocol: once the cephalic vein is accessed, the port catheter, after being flushed and connected to the port chamber, is advanced through the vein into the subclavian vein and toward the right atrium under fluoroscopic guidance using the C-arm in the operating room. A subcutaneous pocket is created in the chest wall for the port, typically above the pectoralis fascia. The catheter is attached to the port chamber, which is placed in the pocket. The port is sutured to the pectoral fascia with non-absorbable sutures for stability. The system is flushed with heparinized saline and checked for aspiration of blood. After achieving hemostasis, the incisions are closed in layers and dressed. The patient is allowed to recover from anesthesia and usually observed for a few hours postoperatively.

**Data Collection and Statistical Analysis:**

For each port placement performed by IR, we recorded the number and roles of staff present and whether one or two consent forms were required. Procedure time was measured from the first incision or puncture to completion of dressing. Procedural complications were recorded during port implantation and the immediate intraprocedural period. Systematic longitudinal follow-up for delayed port-related complications was not included in the study protocol. Consequently, delayed infectious, thrombotic, and mechanical complications were not assessed in the present analysis. Cost data were obtained from the hospital’s finance department for both modalities and included costs related to the consent process, personnel, procedural time, and other associated procedural resources. Costs associated with delayed complications, subsequent treatment, catheter revision, or premature device removal were not incorporated into the cost analysis.

Descriptive statistics were used to summarize the IR cohort, with continuous variables, including procedure time and cost, presented as averages. Surgical procedural and cost data were available only as institutional aggregate averages, without individual patient-level observations or measures of dispersion, and were therefore used as an institutional benchmark rather than as a matched control cohort. Consequently, formal inferential statistical comparisons between IR and surgical procedures, including hypothesis testing and calculation of confidence intervals for between-group differences, were not performed. Comparisons with the surgical benchmark should therefore be interpreted as descriptive and exploratory. Cost and time savings were annualized based on the average number of ports placed per year at our center. No inferential statistical analysis of complication rates was performed.

## 3. Results

### 3.1. Consent Process and Personnel

All 93 patients in the IR group completed a single combined informed consent covering the port placement under local anesthesia. On the other hand, the surgical pathway requires two separate consents, one for the surgical procedure and one for anesthesia, effectively doubling the paperwork and preprocedural consultations for patients. The staffing requirements also differed markedly. A typical surgical port insertion involves four medical professionals: the attending surgeon, an assisting surgeon or surgical nurse, an attending anesthesiologist, and an anesthesia nurse or technician. In our institution, this is the standard minimum for any OR case involving anesthesia. In comparison, an IR-guided port insertion is performed with just two staff members: the interventional radiologist and a radiology technologist. This reduction in personnel not only has cost implications but also scheduling advantages, as coordinating fewer individuals is advantageous. Moreover, omitting anesthesia and sedation with agents such as propofol eliminates the requirement for postoperative recovery in an anesthesia unit. All 93 IR-guided port placements were completed successfully with no intraprocedural complications.

### 3.2. Procedure Time

The time efficiency of the IR approach was higher. Across 93 IR cases, the mean procedure time was approximately 45 min (±10 min). This includes the time for ultrasound guidance, fluoroscopy, and wound closure. For surgical port placements at our center, the average documented operative time was 85 min including both the surgical and anesthesia components. This represents nearly double the duration of an IR case. The longer time in an OR is partly due to the anesthesia induction as well as the meticulous dissection and vessel cut-down process. The 45 min vs. 85 min difference means IR can potentially accommodate almost two port placements in the time surgeons take for one. According to data provided by our institution’s financial department, an average of 188 port implantations are performed annually at our center, encompassing both OR and IR placements. Based on this annual procedural volume, performing all port implantations in the IR suite would save an estimated 234 h of procedure time per year compared with performing all procedures in the operating room.

### 3.3. Cost Analysis

**Figure 2:**
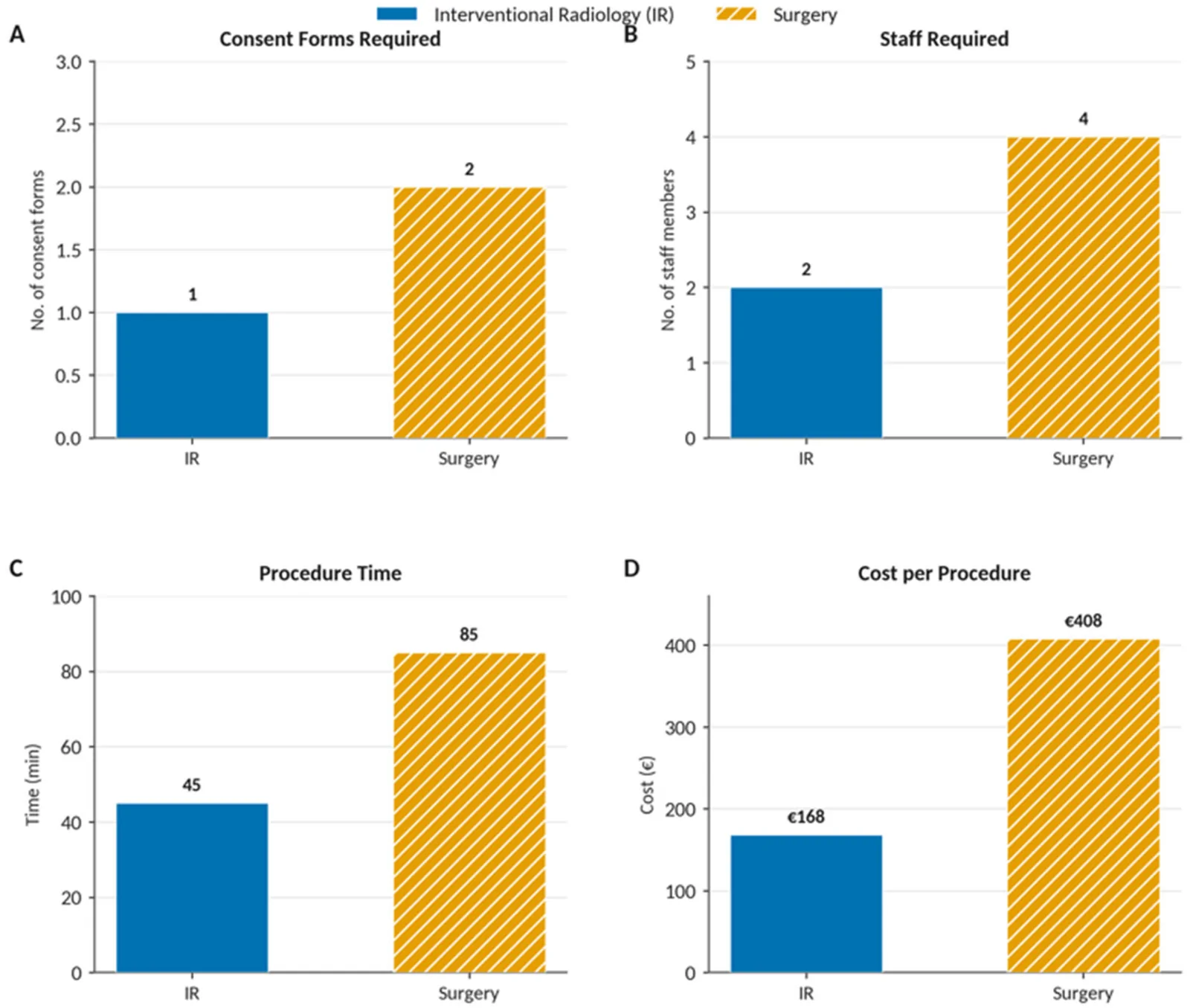
Descriptive comparison of consent requirements, personnel, procedure duration, and cost for IR-performed TIVAPs with institutional aggregate surgical benchmarks. Each pair of bars depicts IR (blue) and Surgery (yellow) values for the specified parameter. (**A**,**B**) The IR technique requires a single consent form and two staff members, whereas the surgical approach necessitates two consent forms and four staff members. (**C**,**D**) The mean procedure time is 45 min for IR compared with 85 min for Surgery, and the average cost per procedure is €168 for IR versus €408 for Surgery.

The direct procedural cost per port placement in the IR suite was substantially lower than in the OR. Based on detailed hospital billing data, an IR TIVAP insertion costs €168.16 on average, whereas a surgical insertion costs €407.90 on average. Figure 2 illustrates the comparison of key resource metrics between the two approaches, including cost and time. The cost for IR is only about 41.2% of the cost in the OR. The estimated mean cost of TIVAP insertion in the IR cohort was 58.8% lower than the institutional aggregate surgical benchmark. Multiplying by our annual volume, this yields nearly €45,000 in savings per year by utilizing IR for port insertions. The cost breakdown revealed that the port device kit and basic supplies were similar between IR and OR. The major differences were due to operating room charges, anesthesia personnel and medication costs, and longer staff time in the OR.

### 3.4. Material and Medication Utilization

The materials used for the port itself, the catheter and chamber kit, sterile drapes, contrast media, suture materials were essentially the same in both settings, and their cost is included in the above analysis. A notable distinction is that in the OR, anesthetic agents such as propofol infusions or intravenous midazolam are routinely administered by the anesthesia team. In our surgical cohort, virtually all patients received some form of sedation or in some cases, general anesthesia. In contrast, in IR, the standard practice was to use local anesthetic with 10 mL of mepivacaine 1% for skin and subcutaneous numbing. Sedation was used sparingly, only when patients requested or required mild sedation, which was achieved with 1 to 2 mg of lorazepam administered orally. No inhaled anesthetic gases or extensive anesthesia circuits were involved. This difference not only reduces cost, but also means IR cases produce less pharmaceutical waste and avoid the environmental impact associated with anesthetic gases. In addition to anesthesia-related supplies, surgical cases required a greater number of surgical disposables, such as those used for electrocautery, whereas IR cases primarily relied on imaging equipment, including sonography.

## 4. Discussion

Our analysis highlights the multifaceted advantages of TIVAP**s** performed by interventional radiologists, particularly in terms of efficiency and observed cost difference. The benefits extend across several dimensions, with the procedure requiring only half the clinical personnel, reducing operative time by approximately 40 min on average, and was associated with nearly 60% lower per-procedure expenditure relative to the institutional surgical benchmark. Importantly, these observed differences should not be interpreted as an intrinsic advantage of interventional radiologists over surgeons. Rather, they primarily reflect differences in the procedural setting and anesthesia strategy, particularly the use of local anesthesia in the angiography suite compared with an operating-room pathway requiring anesthesia support and post-anesthesia recovery.

Both surgeons and interventional radiologists are well equipped to perform TIVAP**s** given their training and technical expertise. However, our study demonstrates that implantation in the angiography suite was associated with lower resource utilization in our institutional setting, particularly over the long term. These findings contrast with a 2009 U.S. study by Sticca et al., which reported higher billed charges for ports placed by interventional radiologists compared with surgically placed ports ($5301 vs. $4552, *p* = 0001) [13]. This discrepancy may partly reflect differences in healthcare systems, billing structures, procedural settings, and anesthesia practices. In our contemporary European setting, cost calculations based on actual resource utilization showed average savings of €239 per port for the IR-led local-anesthesia pathway relative to the institutional surgical benchmark. These findings reflect the resource implications of implementing a streamlined image-guided, local-anesthesia pathway rather than a cost advantage attributable to operator specialty. Importantly, patients entering the IR pathway were considered clinically suitable for implantation under local anesthesia and therefore cannot be assumed to represent the same clinical population as patients undergoing surgical implantation. The surgical data should therefore be regarded as a contextual benchmark rather than a clinically equivalent control group; consequently, our study neither establishes equivalence between the pathways nor determines optimal patient selection for local-anesthesia versus operating-room placement. Notably, Sticca et al. also found no significant difference in short-term complication rates between interventional radiology and surgical groups [13]. Several studies have documented the high success and low complication rates of IR placed ports [6,14,15]. Shah et al. noticed no significant differences in the rate of infection between the angiographic suite and the operation theatre (*p* < 0.743) [16]. Our 0% intraprocedural complication rate in 93 cases underscores the reliability of modern IR techniques. IR operators use real-time imaging to avoid complications, for example, ultrasound guidance eliminates the risk of inadvertent arterial puncture or pneumothorax during venous access. Plumhans et al. found that ultrasound-guided IJV access, as used by interventional radiologists, had significantly fewer arterial punctures and a lower catheter malposition rate than landmark-guided subclavian access [12], reinforcing the technical advantages of the IR approach. Interventional radiologists are specifically trained in radiation safety, and operator exposure is minimized through collimation, low-dose protocols, and protective equipment. Levels of procedural radiation exposure depend on both patient factors such as body habitus and procedure complexity and operator factors such as equipment settings and positioning of staff relative to the X-ray field of view [17]. A study by Lee et al. found that all 133 non-radiologist physicians surveyed reported the dose of a chest radiograph incorrectly, and 77% underestimated radiation exposure in imaging examinations [18]. Radiologists by contrast are generally more aware of these aspects and consistently apply the ALARA principle in clinical practice [17]. For port implantation, patient exposure is not expected to differ between surgical and IR approaches since in both cases, fluoroscopy is only used to confirm catheter position. The difference lies in the expertise of the operator and the radiation protection environment [19].

During the COVID-19 pandemic, we observed an increase in the number of TIVAP**s** inserted by interventional radiologists, driven by the implementation of numerous safety regulations especially in the operation theatre and the ongoing rise in staff illness-related absences and quarantine due to COVID-19. This phenomenon was also observed in the UK but not in all IR centers. Zhong et al. reported that of six centres in the UK, three experienced a 38–145% increase while the other three experienced a 25–80% decrease, and overall, there was a 0.5% caseload reduction [20]. The adaptability of IR due to providing line services and bedside procedures to support critical care services might have contributed to the increased demand in some centres during COVID-19 [20,21]. In our center, performing more interventional radiology procedures helped decrease the workload in the operation theatres to align with the safety regulations during the pandemic.

The limitations of this study include its single-center design and the absence of an accurately matched surgical cohort. Comparisons with the surgical pathway were based on aggregate institutional database averages rather than patient-level matched controls. Hence, the surgical data should be considered an institutional benchmark rather than a matched control cohort. Allocation to the IR or surgical pathway was determined by routine clinical scheduling rather than randomization, introducing the potential for selection bias. Differences in patient characteristics, procedural complexity, anesthesia requirements, operator experience, and other unmeasured factors could not be assessed and may have contributed to the observed differences between the two pathways. Moreover, because patients entering the IR pathway were considered clinically suitable for implantation under local anesthesia, this study cannot determine the optimal criteria for triaging patients between local-anesthesia and operating-room pathways. Furthermore, because surgical data were available only as aggregate institutional averages without measures of variability, formal inferential statistical comparisons between the two pathways could not be performed. Accordingly, observed differences in procedural time and costs should be interpreted as descriptive and hypothesis-generating rather than as evidence of superiority of one approach. Outcome assessment was restricted to the immediate intraprocedural period. Therefore, delayed infectious, thrombotic, and mechanical complications were not systematically captured, precluding comparison of short- and long-term complication rates between the IR and surgical pathways and limiting conclusions regarding overall clinical safety. The present findings should therefore be interpreted primarily in terms of operational efficiency and direct procedural resource utilization. Finally, the cost analysis reflects the resource structure of a single European institution, and the annualized estimates are based on a hypothetical scenario in which the institution’s annual port implantation volume is managed through the IR pathway. Consequently, the economic findings may not be directly generalizable to other healthcare systems or institutions with different organizational structures. Future prospective studies incorporating patient-level surgical comparators and standardized 30- or 90-day follow-up are needed to validate the observed differences in resource utilization, costs, and clinical outcomes.

## 5. Conclusions

Performing TIVAP implantations in the angiography suite rather than the operating room highlights the potential contribution of interventional radiology to more resource-efficient and sustainable healthcare delivery. In our institution, the estimated mean procedural cost of the IR pathway was 58.8% lower than the institutional aggregate surgical benchmark, while the IR workflow required two staff members compared with four in the surgical pathway. Avoidance of general anesthesia may also reduce pharmaceutical consumption, anesthetic gas emissions, and the use of disposable materials, potentially decreasing the environmental footprint of the procedure. By conserving financial, material, and human resources, an IR-based workflow may allow operating room capacity to be redirected toward more complex surgical cases.

## Data Availability

The raw data supporting the conclusions of this article will be made available by the authors on request.

## Abbreviations

The following abbreviations are used in this manuscript:

IR	Interventional Radiology
OR	Operating Room
COVID-19	Coronavirus Disease 2019
TIVAP	totally implantable venous access port
